# Supplementary material for: An open-source framework for large-scale, flexible evaluation of biomedical text mining systems
Source: J Biomed Discov Collab. 2008 Jan 29;3:1. doi: 10.1186/1747-5333-3-1 (PMC2276192; doi:10.1186/1747-5333-3-1)
Supplement: Additional file 1 — Evaluation platform source code. The additional file contains the software evaluation framework discussed in this paper. The most current release can be downloaded from SourceForge.net [1]. See the accompanying README file for instructions on installation and use. Also included in the distribution is a collection of UIMA wrappers for some commonly used BioNLP tools and annotated corpora. [file 1747-5333-3-1-S1.zip › bionlp_uima_repository-1.0.1/docs/api/src-html/edu/uchsc/ccp/uima/ae/util/filter/ClassMentionTypeFilter_AE.html]

```
001    package edu.uchsc.ccp.uima.ae.util.filter;
002    
003    import java.util.ArrayList;
004    import java.util.Iterator;
005    import java.util.List;
006    
007    import org.apache.uima.analysis_engine.ResultSpecification;
008    import org.apache.uima.analysis_engine.annotator.AnnotatorConfigurationException;
009    import org.apache.uima.analysis_engine.annotator.AnnotatorContext;
010    import org.apache.uima.analysis_engine.annotator.AnnotatorContextException;
011    import org.apache.uima.analysis_engine.annotator.AnnotatorInitializationException;
012    import org.apache.uima.analysis_engine.annotator.AnnotatorProcessException;
013    import org.apache.uima.analysis_engine.annotator.JTextAnnotator_ImplBase;
014    import org.apache.uima.jcas.JCas;
015    
016    import edu.uchsc.ccp.uima.annotation.CCPTextAnnotation;
017    import edu.uchsc.ccp.uima.mention.CCPClassMention;
018    
019    /**
020     * removes all annotations that are not a specific class mention
021     * @author williamb
022     *
023     */
024    public class ClassMentionTypeFilter_AE extends JTextAnnotator_ImplBase {
025    
026        public static final String PARAM_CLASS_MENTION_TYPES_TO_KEEP_LIST = "ClassMentionTypesToKeepList";
027            
028        private List<String> classMentionTypesToKeep;
029        
030        @Override
031        public void initialize(AnnotatorContext ac) throws AnnotatorInitializationException, AnnotatorConfigurationException {
032            /* read in input parameters, and initialize a list of class mention types to keep */
033            try {
034                String[] classMentionTypes = (String[]) ac.getConfigParameterValue(PARAM_CLASS_MENTION_TYPES_TO_KEEP_LIST);
035                classMentionTypesToKeep = new ArrayList<String>();
036                for (String type : classMentionTypes) {
037                    classMentionTypesToKeep.add(type);
038                }
039            } catch (AnnotatorContextException e) {
040                e.printStackTrace();
041            }
042            super.initialize(ac);
043        }
044        
045        
046        /**
047         * cycle through all annotations and set the annotation set
048         */
049        public void process(JCas jcas, ResultSpecification arg1) throws AnnotatorProcessException {
050            
051            List<CCPTextAnnotation> annotationsToRemove = new ArrayList<CCPTextAnnotation>();
052            
053            Iterator annotIter = jcas.getJFSIndexRepository().getAnnotationIndex(CCPTextAnnotation.type).iterator();
054    
055            while (annotIter.hasNext()) {
056                Object possibleAnnot = annotIter.next();
057                if (possibleAnnot instanceof CCPTextAnnotation) {
058                    CCPTextAnnotation ccpTA = (CCPTextAnnotation) possibleAnnot;
059                    CCPClassMention ccpCM = ccpTA.getClassMention();
060                    String classMentionType = ccpCM.getMentionName();
061                    if (!classMentionTypesToKeep.contains(classMentionType)) {
062                        annotationsToRemove.add(ccpTA);
063                    }
064                } else {
065                    System.err.println("WARNING -- AnnotatorFilter_AE: CCPTextAnnotation expected but instead got "
066                            + possibleAnnot.getClass().getName());
067                }
068            }
069            
070            /* now remove annotations that had class mention types not in the classMentionTypesToKeep list */
071            for (CCPTextAnnotation ccpTA : annotationsToRemove) {
072                ccpTA.removeFromIndexes();
073                ccpTA = null;
074            }
075    
076        }
077    
078        
079    
080    }
```
